# Author Correction: A synthetic nanobody targeting RBD protects hamsters from SARS-CoV-2 infection

**DOI:** 10.1038/s41467-022-32074-w

**Published:** 2022-07-27

**Authors:** Tingting Li, Hongmin Cai, Hebang Yao, Bingjie Zhou, Ning Zhang, Martje Fentener van Vlissingen, Thijs Kuiken, Wenyu Han, Corine H. GeurtsvanKessel, Yuhuan Gong, Yapei Zhao, Quan Shen, Wenming Qin, Xiao-Xu Tian, Chao Peng, Yanling Lai, Yanxing Wang, Cedric A. J. Hutter, Shu-Ming Kuo, Juan Bao, Caixuan Liu, Yifan Wang, Audrey S. Richard, Hervé Raoul, Jiaming Lan, Markus A. Seeger, Yao Cong, Barry Rockx, Gary Wong, Yuhai Bi, Dimitri Lavillette, Dianfan Li

**Affiliations:** 1 State Key Laboratory of Molecular Biology, CAS Center for Excellence in Molecular Cell Science, Shanghai Institute of Biochemistry and Cell Biology, Chinese Academy of Sciences (CAS), Shanghai, China; 2 University of CAS, Beijing, China; 3 CAS Key Laboratory of Molecular Virology & Immunology, Institut Pasteur of Shanghai CAS, Shanghai, China; 4 CAS Key Laboratory of Pathogenic Microbiology and Immunology, Institute of Microbiology, Center for Influenza Research and Early-warning (CASCIRE), CAS-TWAS Center of Excellence for Emerging Infectious Diseases (CEEID), CAS, Beijing, China; 5 Erasmus Laboratory Animal Science Center, Erasmus University Medical Center, Rotterdam, Netherlands; 6 European Research Infrastructure on Highly Pathogenic Agents (ERINHA-AISBL), Paris, France; 7 Department of Viroscience, Erasmus University Medical Center, Rotterdam, Netherlands; 8 National Facility for Protein Science in Shanghai, Shanghai Advanced Research Institute (Zhangjiang Laboratory), CAS, Shanghai, China; 9 Institute of Medical Microbiology, University of Zurich, Zurich, Switzerland; 10 Département de microbiologie-infectiologie et d’immunologie, Université Laval, Québec, QC Canada; 11 Pasteurien College, Soochow University, Jiangsu, China

**Keywords:** X-ray crystallography, SARS-CoV-2

Correction to: *Nature Communications* 10.1038/s41467-021-24905-z, published online 30 July 2021

The original version of the Supplementary Information associated with this Article included an incorrect Supplementary Data 1 file, in which sequences were duplicated. The HTML has been updated to include a corrected version of Supplementary Data 1; the original incorrect version of Supplementary Data 1 can be found as Supplementary Information associated with this Correction.

## Supplementary information

Supplementary Data 1

