# Supplementary material for: A synthetic nanobody targeting RBD protects hamsters from SARS-CoV-2 infection
Source: Nat Commun. 2021 Jul 30;12:4635. doi: 10.1038/s41467-021-24905-z (PMC8324831; doi:10.1038/s41467-021-24905-z)
Supplement: Supplementary file 1 — Supplementary Information [file 41467_2021_24905_MOESM1_ESM.pdf]

A synthetic nanobody targeting RBD protects hamsters from SARS-CoV-2 infection

Tingting Li[1,2,#], Hongmin Cai[1,2,#], Hebang Yao[1,2,#], Bingjie Zhou[2,3,#], Ning Zhang[4,#], Martje Fentener van Vlissingen[5,8], Thijs Kuiken[6,8], Wenyu Han[1,2], Corine H. GeurtsvanKessel[6,8], Yuhuan Gong[2,4], Yapei Zhao[2,3], Quan Shen[4], Wenming Qin[7], Xiaoxu Tian[7], Chao Peng[7], Yanling Lai[1,2], Yanxing Wang[1], Cedric A.J. Hutter[8], Shu-Ming Kuo[3], Juan Bao[1], Caixuan Liu[1,2], Yifan Wang[1,2], Audrey S. Richard[8], Hervé Raoul[8], Jiaming Lan[3], Markus A. Seeger[9], Yao Cong[1], Barry Rockx[6,8], Gary Wong[3,10*], Yuhai Bi[2,4,*], Dimitri Lavillette[3,11*], Dianfan Li[1,*]

Supplementary Table 1

Supplementary Figures 1-14

Supplementary Table 1. Cryo-EM data collection and reconstruction statistics.

	MR3-Spike complex
**Data collection**	
EM equipment	Titan Krios
Voltage (kV)	300
Detector	K2 Summit
Pixel size (Å)	1.02
Electron dose (e^-/Å^2)	49.6
Exposure time (s)	6.45
Frames	43
Defocus range (μm)	-0.8 to -2.5
**Reconstruction**	
Softwares	Relion 3.1 & cryoSPARC
Final particles	34,243
Symmetry	C1
Final overall resolution (Å)	6.25

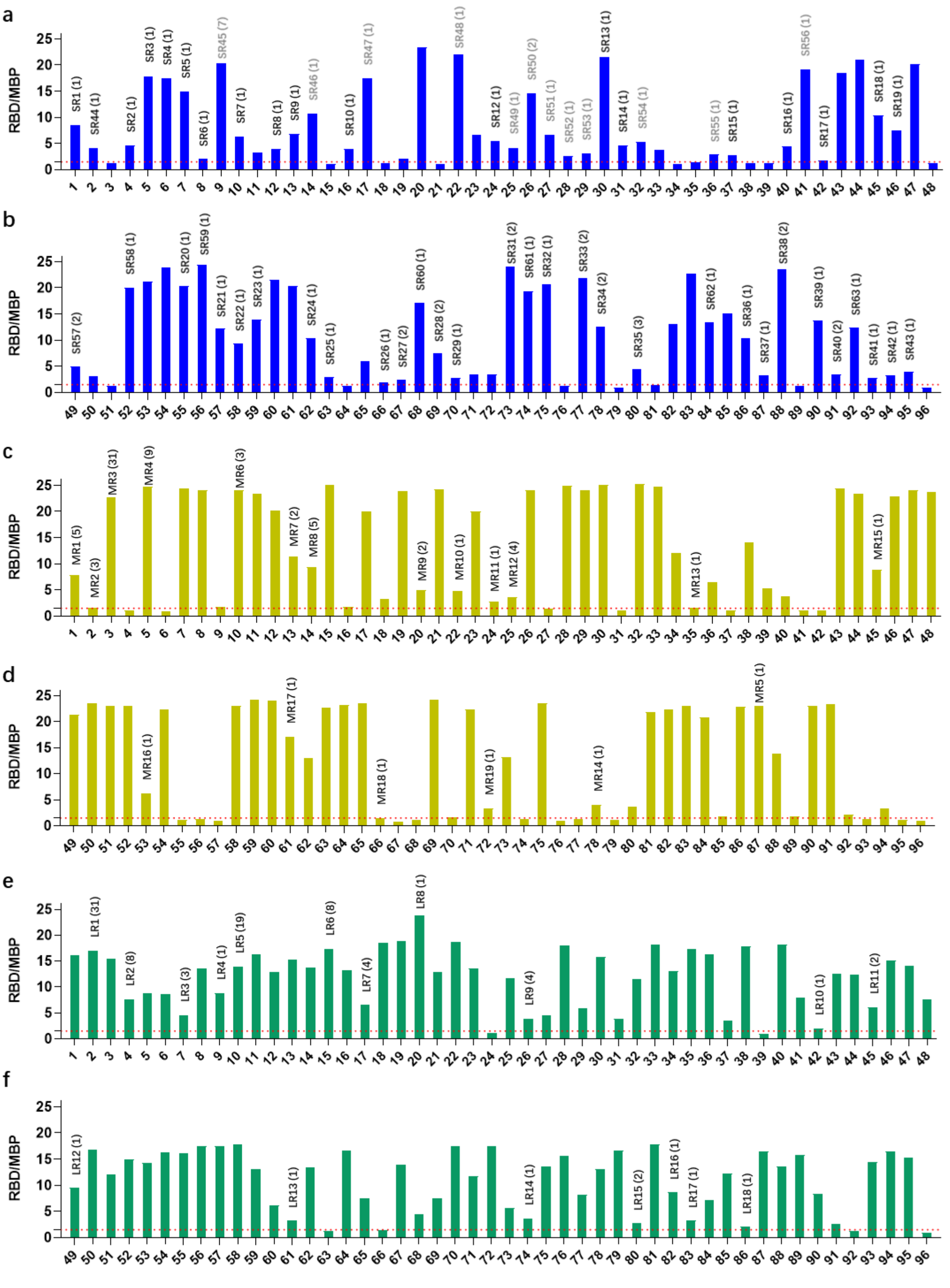

Supplementary Fig. 1. Identification of RBD binders using ELISA. (**a, b**) Results for the Concave library. (**c, d**) Results for the Loop library. (**e, f**) Results for the Convex library. The ratio between the ELISA signal (A_{650}) of wells with the RBD and of wells with the unrelated maltose-binding protein (MBP) is plotted. The signal for MBP is typically between 0.04-0.09. A red dashed line guides the cut-off at a ratio of 1.5. Unique clones are labeled with the redundancy shown in brackets.

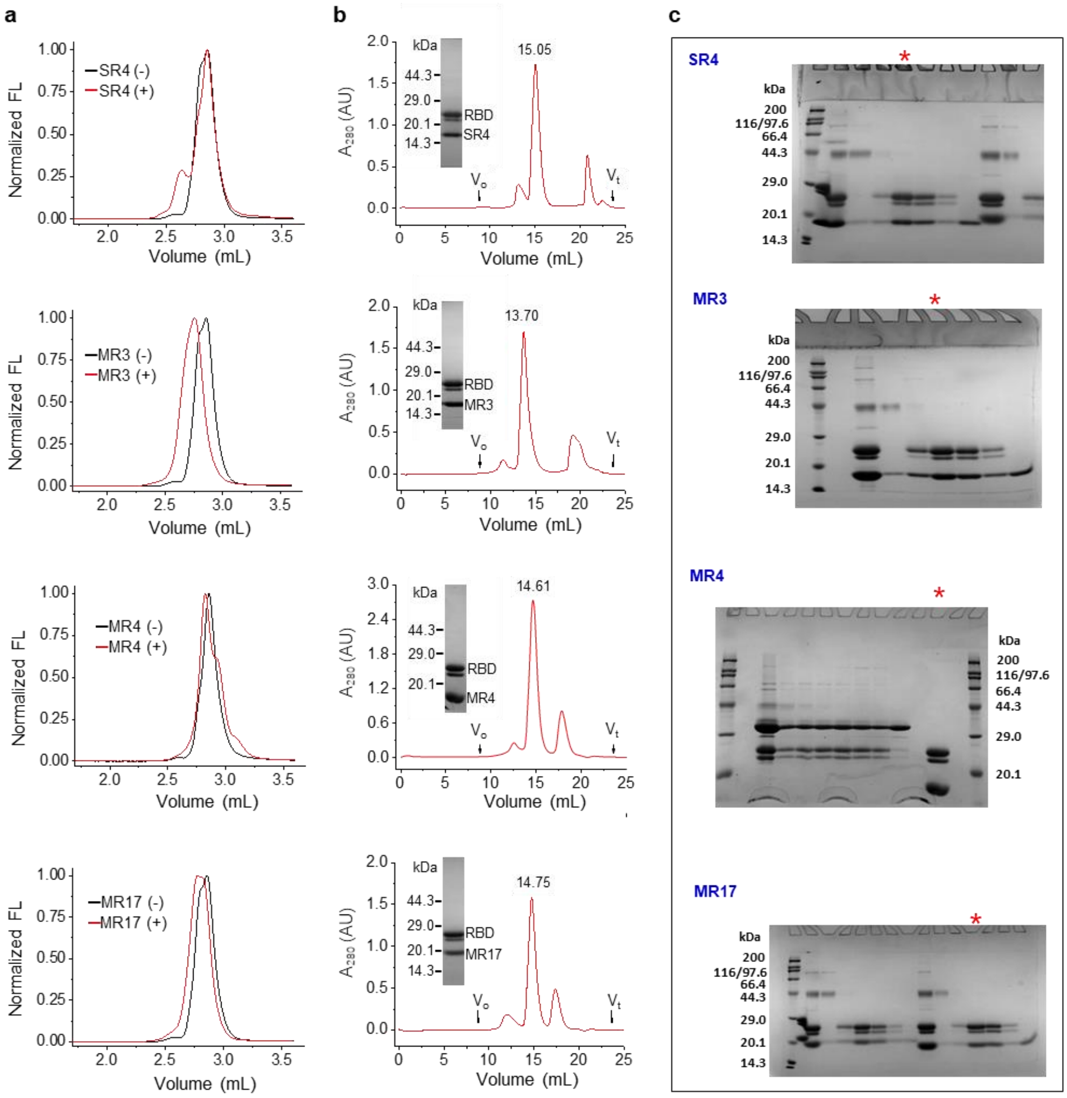

Supplementary Fig. 2. Characterization and purification of sybody-RBD complexes. (**a**) Fluorescence-detector size exclusion chromatography (FSEC) of the RBD in the absence (black, -) and presence (red, +) of crude periplasmic extract from sybody clones. Biotinylated RBD was fluorescently labeled through binding to streptavidin that was conjugated with an amine-reactive fluorescein variant. The concentration of RBD was 0.5 µM. Fluorescence (Ex. 482 nm, Em. 508 nm) (FL) was normalized before plotting. The extent of peak shift follows the order of SR4<MR4<MR17<MR3. The fluorescence trace before the void volume (V_o, 1.78 mL) is not shown. (**b**) Preparative size exclusion chromatography of the indicated sybody-RBD complexes. SDS-PAGE images of the main-peak fraction for all four sybodies are shown in the inset. Numbers label the elution volume for the main peak. AU, absorbance unit. Data are from a single experiment. (**c**) Full scans of the gel image in **b** (inset). An asterisk indicate the lane shown in **b**.

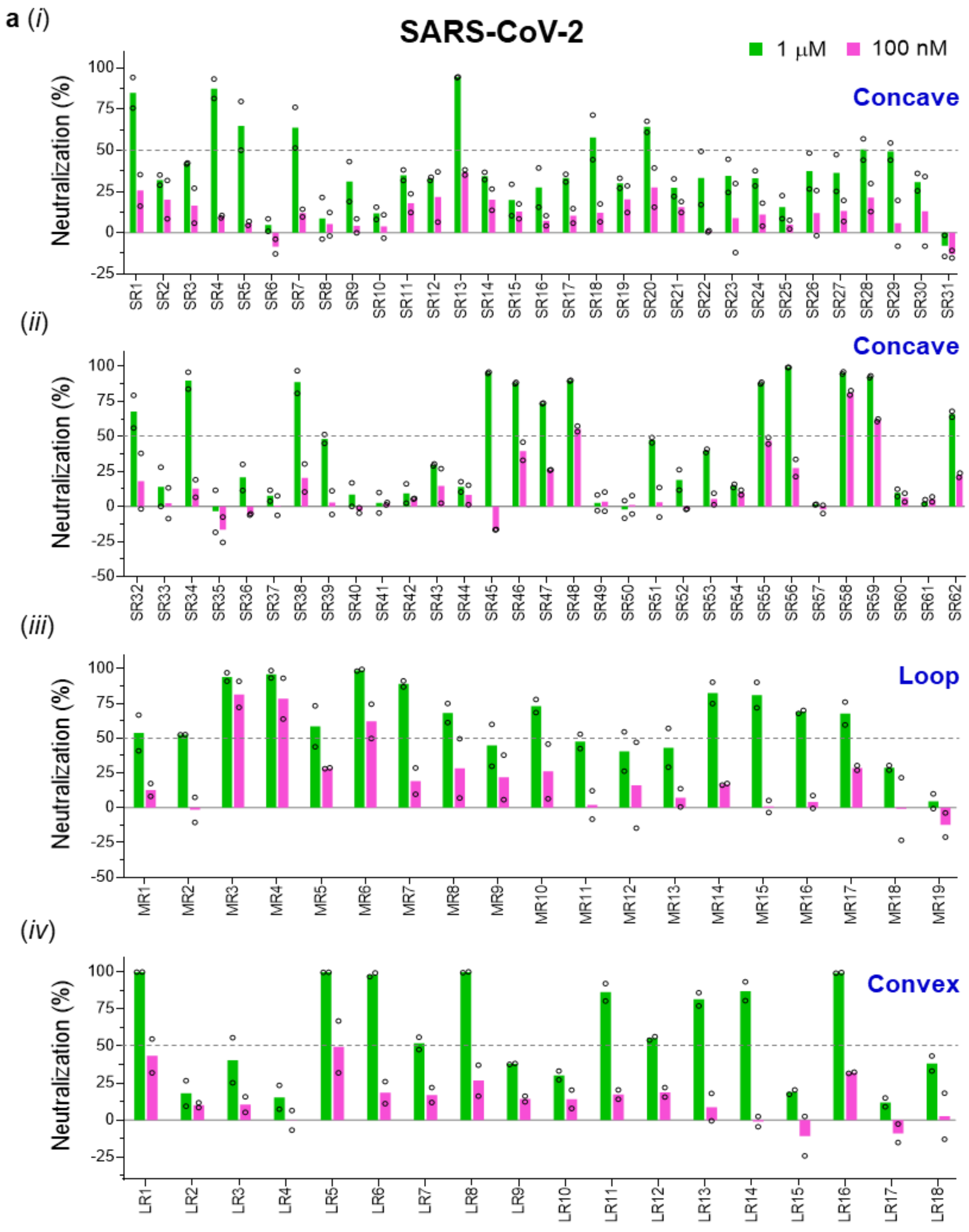

Supplementary Fig. 3. To be continued on the next page.

Supplementary Fig. 3. Continued from the previous page.

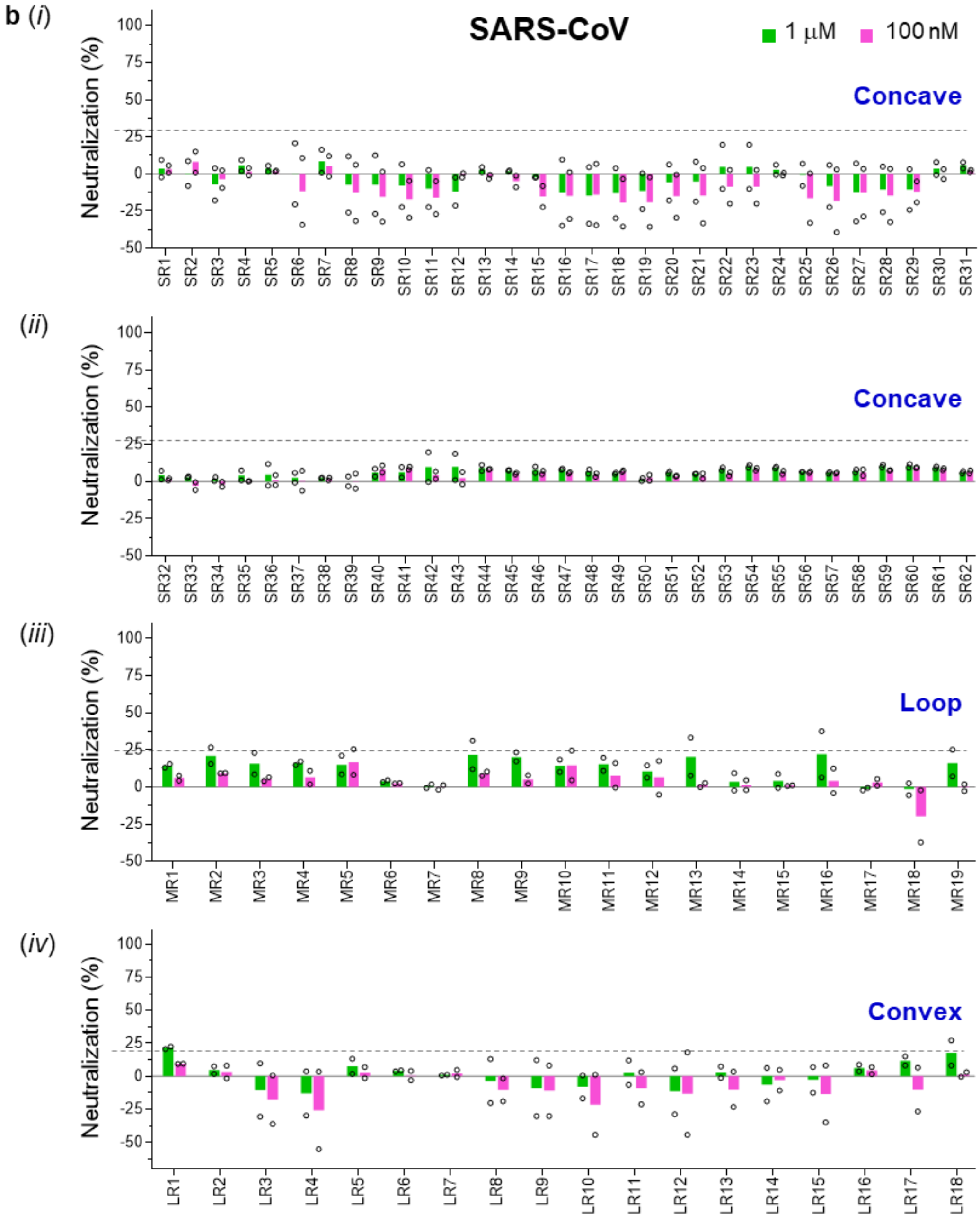

Supplementary Fig. 3. Neutralization activity of 99 sybodies. (**a**) Neutralization assay results for SARS-CoV-2 pseudovirus. (**b**) Neutralization assay results for SARS-CoV pseudovirus. VeroE6-hACE2 cells were infected with a premix of pseudotypes and sybodies at 1 μM (green) and 100 nM (magenta). Infectivity was measured after 3 days using FACS and the percentage of neutralization was calculated for each sybody. Average of data from two independent experiments are plotted. Source data are provided as a Source Data file.

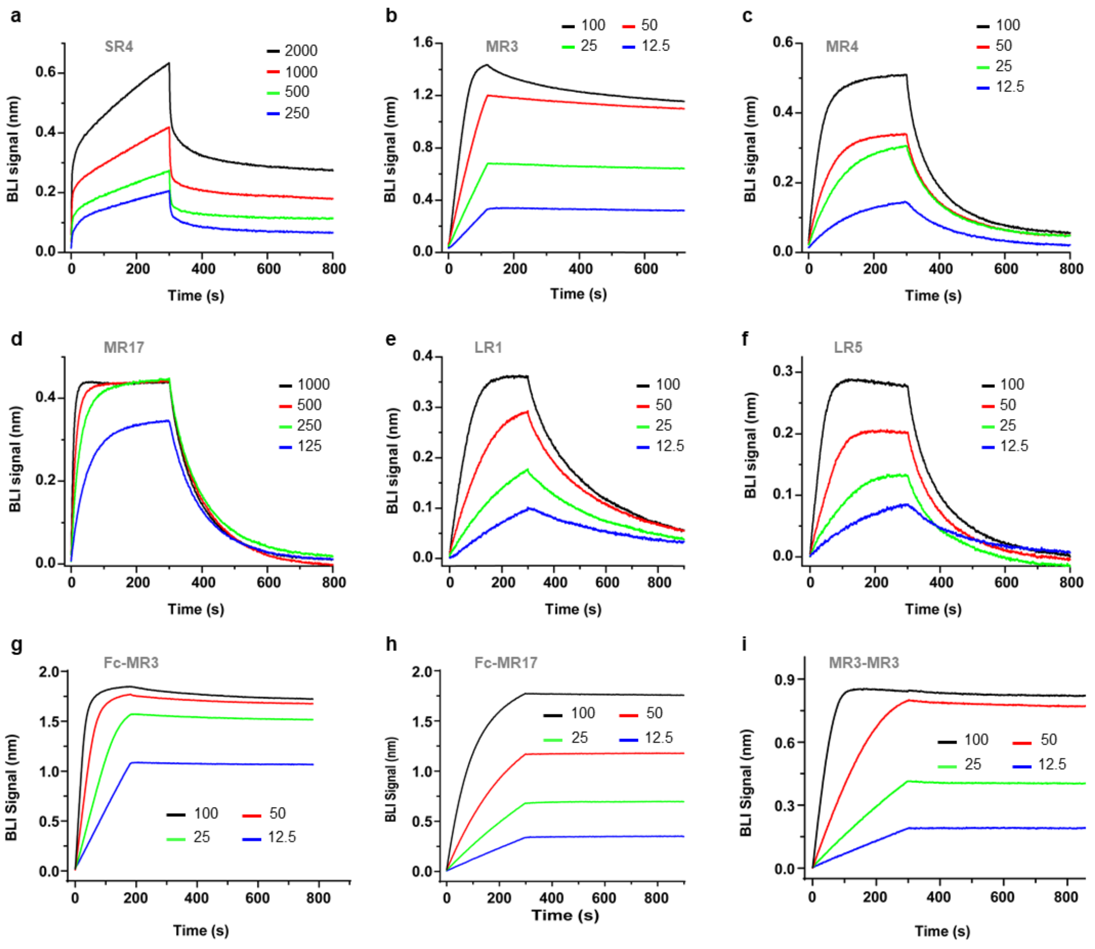

Supplementary Fig. 4. Kinetics for sybody-RBD binding. (**a-i**) Biotinylated RBD immobilized on a streptavidin-coated sensor was titrated with various concentrations (nM) of sybodies as indicated by the color-coding. Bio-layer interferometry (BLI) data were fitted with a 1:1 stoichiometry.

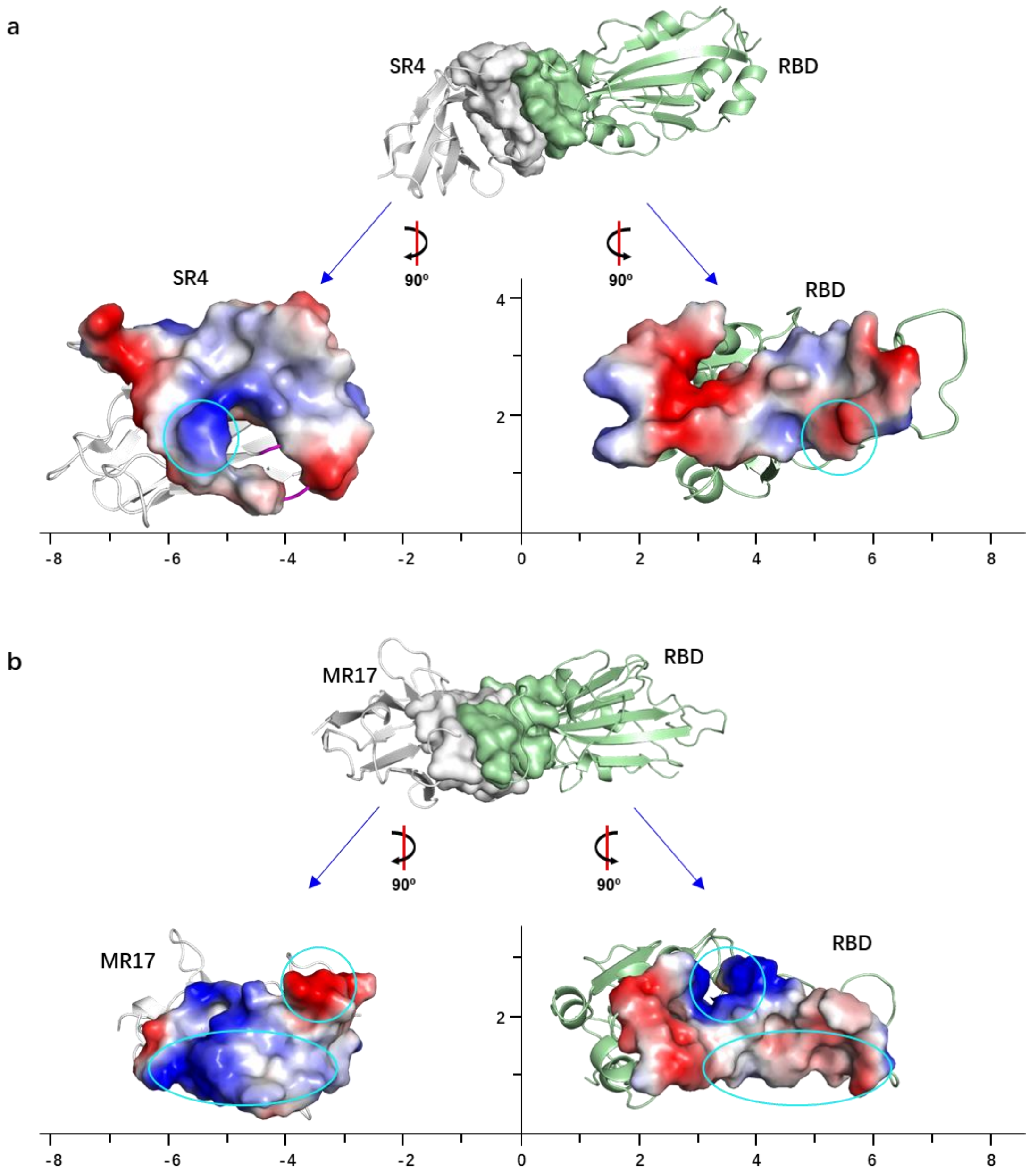

Supplementary Fig. 5. Electrostatic complementarity of the sybody-RBD binding surface. (**a, b**) 'Open-book' view of molecular electrical potential surfaces of the interface between the RBD (pale green) and SR4 (white) (**a**) and between the RBD (pale green) and MR17 (white) (**b**). The electrical potential maps were calculated by Adaptive Poisson-Boltzmann Solver (APBS) [1] built-in in PyMol and drawn with positive potential in blue and negative potential in red. The unitless ruler guides the view of the relative distances between the opened surface pairs. Cyan circles highlight electrostatic complementarity. RBD, receptor-binding domain.

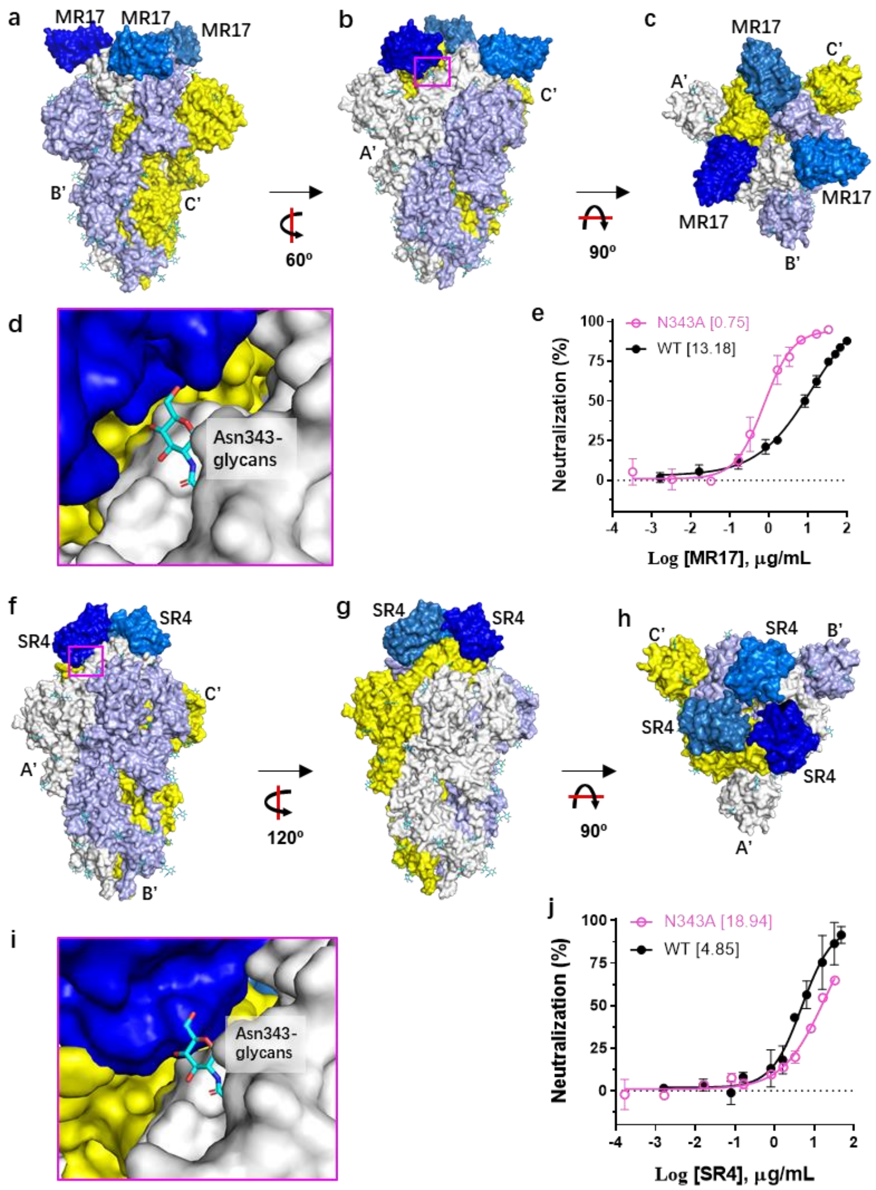

Supplementary Fig. 6. SR4 and MR17 may bind to the SARS-CoV-2 S RBD in the 'closed' conformation. (**a-d**) Aligning the MR17-RBD with the closed conformation (PDB ID 6VXX)[2] of SARS-CoV-2 S. No obvious clashes are observed except for the Asn343-linked glycans (**d**). (**e**) Neutralization assay of MR17 using pseudovirus bearing the wild-type (WT) Spike (closed black circle) or the N343A Spike (open magenta circle). (**f-j**) Aligning the SR4-RBD with the closed conformation (PDB ID 6VXX)[2] of SARS-CoV-2 S. No obvious clashes are observed except for the Asn343-linked glycans (**i**). (**j**) Neutralization assay of SR4 using pseudovirus bearing the wild-type (WT) Spike (closed black circle) or the N343A Spike (open magenta circle). The three identical chains (protomers A', B', and C') of S are colored yellow, white, and pale blue. Sybodies are colored blue. Glycan chains are shown as cyan sticks. **d**/**i** shows the expanded view of the area in the magenta box in **b**/**f**. The neutralization data for the WT are from **Fig. 1b**. IC_{50} values (μg mL^{-1}) are shown in square brackets in **e**/**j**. In **e** and **j**, mean ± standard deviation are plotted (n=3 independent experiments). Error bars are omitted where, in rare cases, available data points are less than three due to experimental design on concentration replicates. Source data for **e** and **j** are provided as a Source Data file.

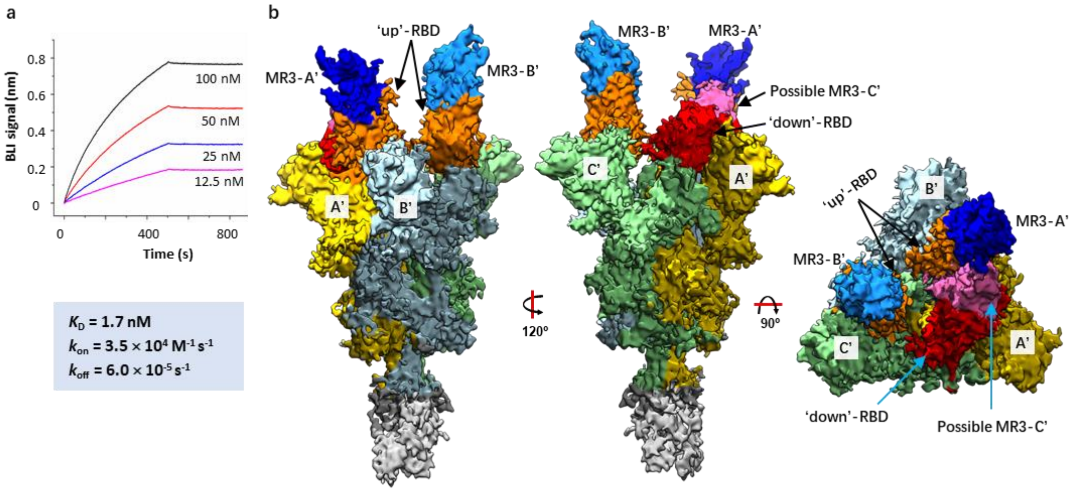

Supplementary Fig. 7. Binding and structural analysis suggest MR3 engage S at the RBM site. (**a**) Binding kinetics between MR3 and S. BLI profile was recorded using immobilized MR3 and S as analyte at indicated concentrations. (**b**) The cryo-EM map of the MR3-Spike complex. A', B', and C' denote the three S protomers. MR3-A' (dark blue), MR3-B' (marine blue), and MR3-C' (pink) label MR3-accomodating densities on Protomers A', B', and C', respectively. Two 'up'-RBDs (orange) and one 'down'-RBD (red) are appropriately labeled. BLI, biolayer interferometry; RBD, receptor-binding domain.

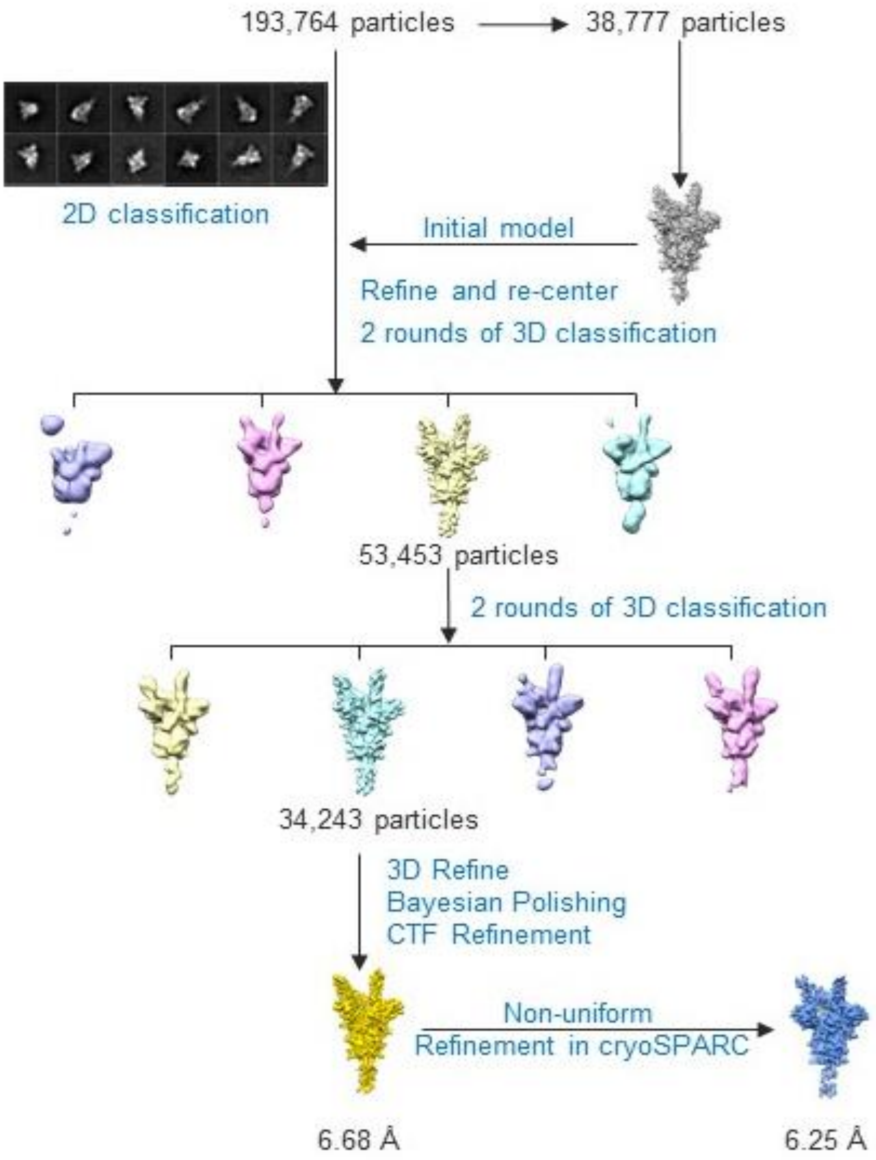

Supplementary Fig. 8. Cryo-EM data processing procedure for the MR3-Spike complex.

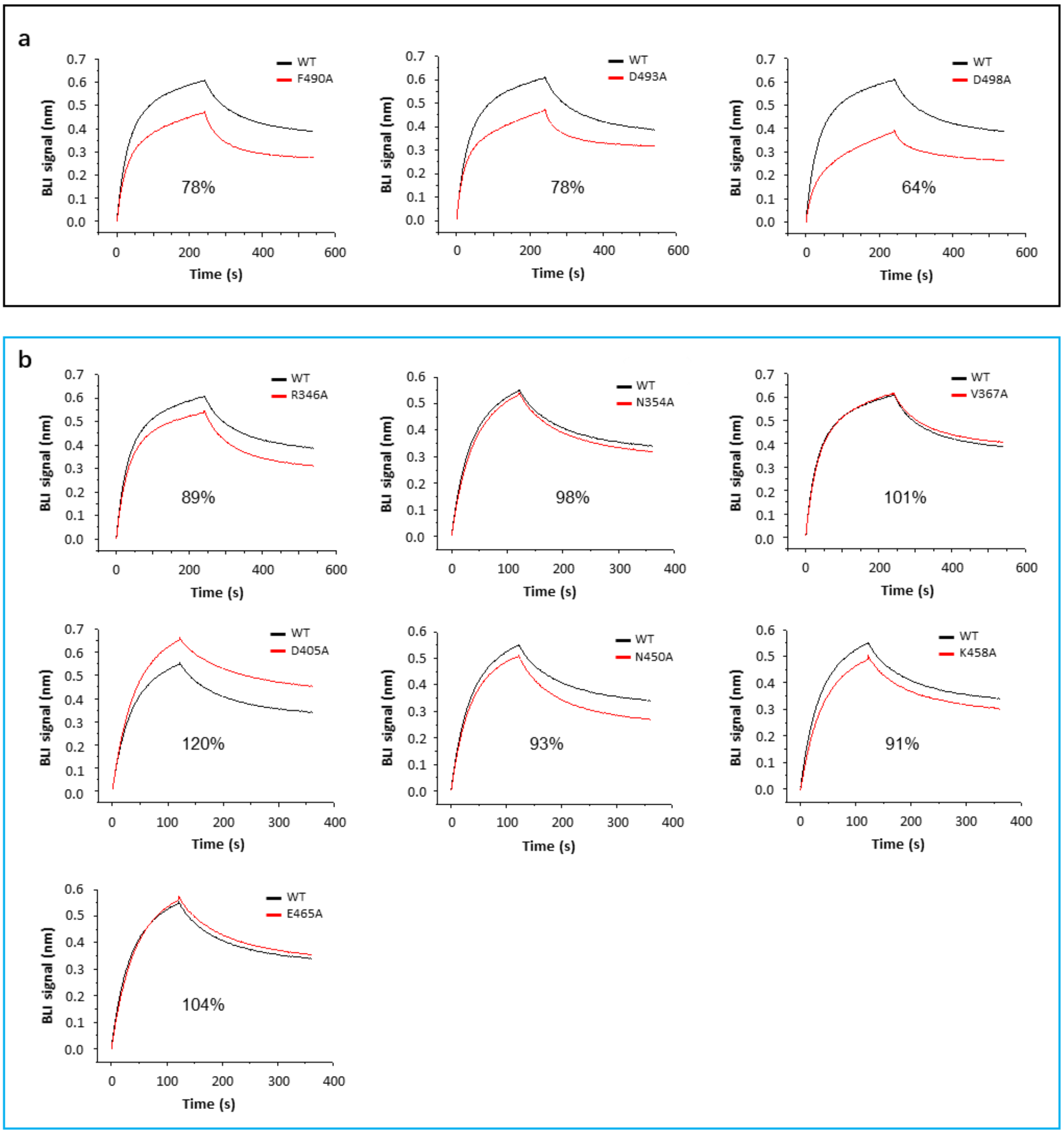

Supplementary Fig. 9. BLI profile for the binding between RBD mutants and MR3. BLI assays were carried out with MR3 immobilized and RBD wild-type (WT, black) or RBD mutants (red) as the analyte. (**a**) RBD mutants displaying 25-80% of BLI signal of the wild-type. (**b**) RBD mutants displaying a similar binding profile as the wild-type. Percentage BLI signal of the wild-type (100%) is indicated in each panel. Mutants showing <25% BLI signal are shown in **Fig. 3i**. Binding signal as percentage to the WT is indicated in each panel. BLI, biolayer interferometry; RBD, receptor-binding domain.

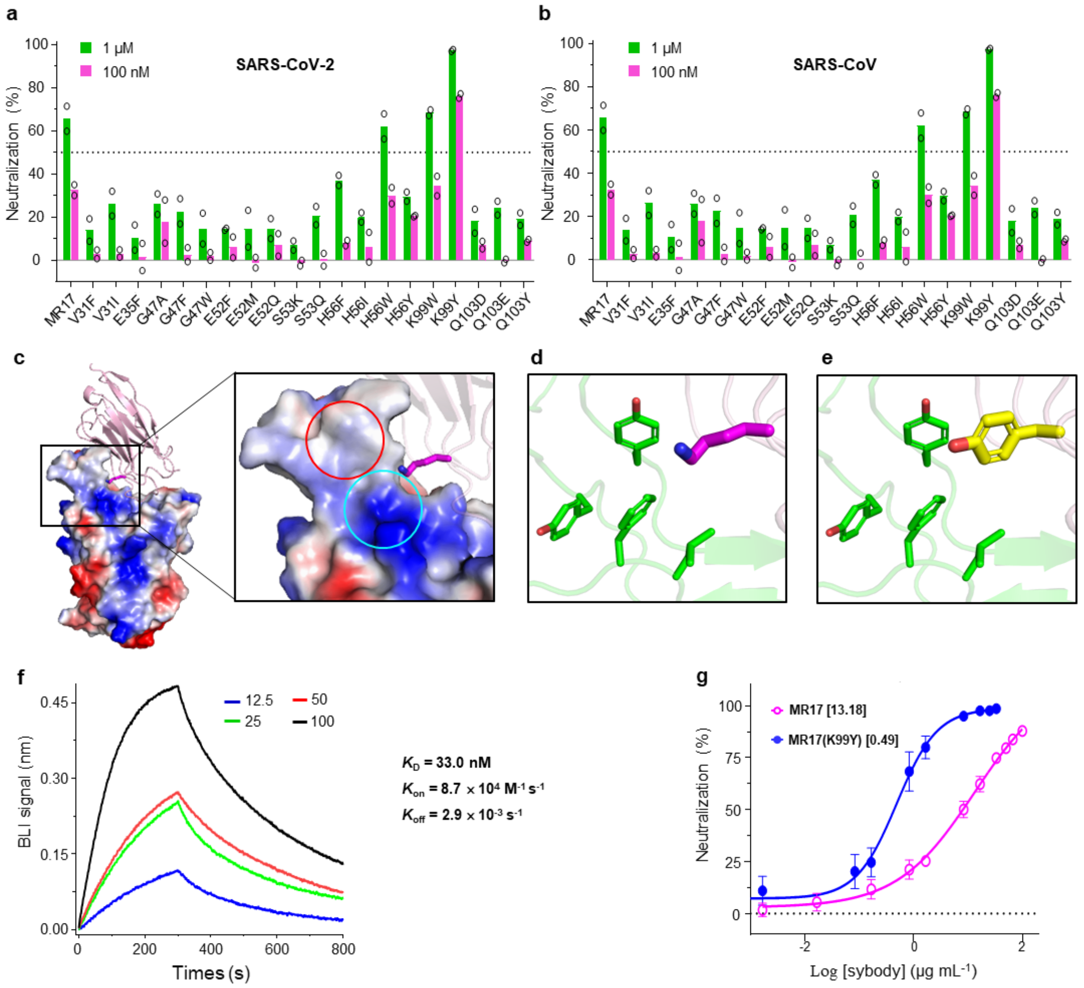

Supplementary Fig. 10. Structure-based design of an MR17 mutant (MR17m) with improved affinity and potency. (**a**, **b**) Neutralization assay for SARS-CoV-2 (**a**) or SARS-CoV (**b**) pseudotypes by the wild-type MR17 sybody and the 20 rationally designed single-mutants (See **Methods**). Sybody concentrations were used at 1 μM (green) and 100 nM (magenta). Data are from two independent experiments. (**c, d**) The rationale for the design of K99Y. The positively charged Lys99 (magenta) pokes to an area (boxed) that contains a hydrophobic patch (red cycle) and a positively charged surface (cyan cycle). Electrostatic repel and hydrophobic mismatch would make Lys99 unfavorable at this position. According to the original library design, Lys99 was unvaried[3], meaning that Lys99 was not selected and hence offering opportunities for optimization. (**e**) The K99Y (yellow) mutation fits the hydrophobic microenvironment well, as revealed by the crystal structure of MR17m (**Table 1**). In **d** and **e**, RBD residues are colored green. (**f**) Binding kinetics of MR17m binding to RBD. Biolayer interferometry (BLI) signals were recorded under indicated MR17m concentrations (nM). (**g**) Comparison of neutralization activity of MR17 (open magenta circle) and MR17m (closed blue circle). IC_{50} values (μg mL^{-1}) for SARS-Cov-2 are indicated in brackets. Data for MR17 are from **Fig. 1b**. In **a** and **b**, average of data from two independent experiments are plotted. In **g,** mean ± standard deviation are plotted (n=3 independent experiments). Error bars are omitted where, in rare cases, available data points are less than three due to experimental design on concentration replicates. Source data for **a**, **b**, and **g** are provided as a Source Data file.

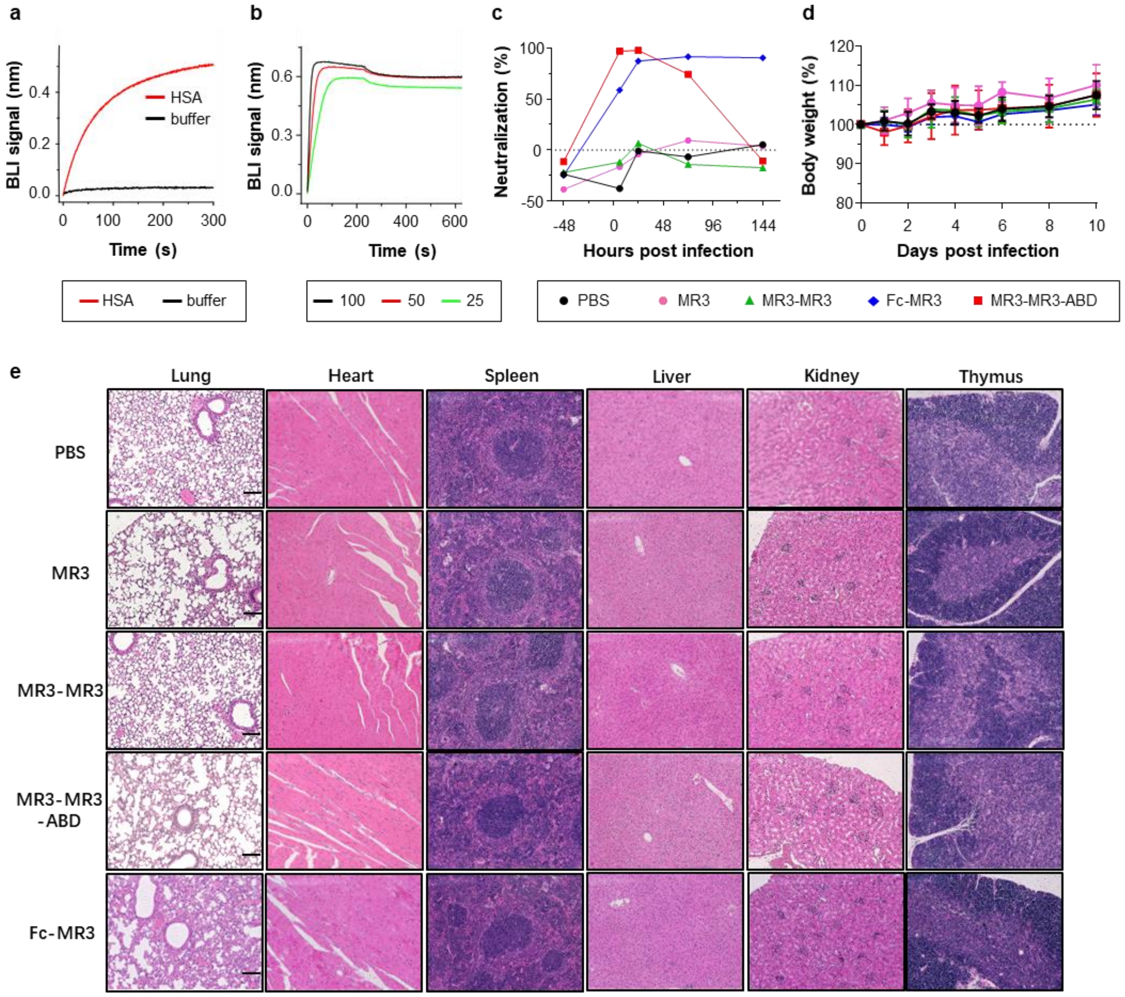

Supplementary Fig. 11. Characterization of MR3-MR3-ABD and evaluation of *in vivo* stability and toxicity of nanobodies. (**a, b**) BLI binding assays show that MR3-MR3-ABD bind to human serum albumin (HSA) (**a**) and RBD (**b**). RBD-coated sensors were incubated with 200 nM of HSA (red, **a**) or buffer (black, **a**), or indicated concentrations of MR3-MR3-ABD (**b**) for single monitoring. (**c**) Neutralization activity of sera from mice injected with sybodies. Sera were collected from mice injected with sybodies MR3 (magenta circle), MR3-MR3 (green triangle), MR3-MR3-ABD (red square), Fc-MR3 (blue diamond), or PBS (black circle) at the indicated time points. For neutralization assay, sera were preincubated with SARS-CoV-2 pseudovirus for 1 h before infection at 1/200 dilution. The infection rates on VeroE6-hACE2 were measure by FACS 3 days post-infection. Data are from a representative of two biologically independent samples. (**d**) Bodyweight changes. The body weight data are presented as means ± the standard deviation of mice in each group (n= 4 biologically independent samples). No significant differences are observed. Color coding of the nanobodies are the same as in **c**. (**e**) Representative (and reproducible in all 4 mice) histopathology of the lungs, heart, liver, spleen, lungs, kidney, and thymus for the different sybodies injected. On day 3, the organs were collected, fixed, sliced, and stained with hematoxylin and eosin. The images and areas of interest are magnified 100×. Bars indicate 100 μm. Source data for **c** and **d** are provided as a Source Data file. ABD, albumin-binding domain; FACS, fluorescence-activated cell sorting; HSA, human serum albumin; PBS, phosphate-buffered saline.

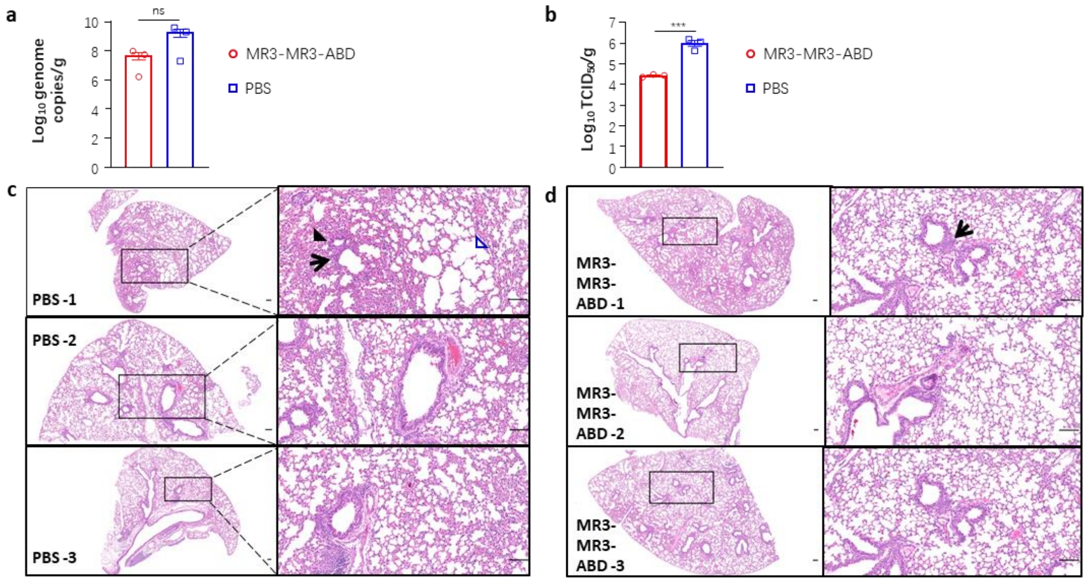

e

Group	alveolar lesions		bronchial lesions		vascular lesions		score
	wall thickening, inflammatory cell infiltration	diffuse alveolar hemorrhage, serous exudation	necrosis	inflammatory cell infiltration	necrosis	perivascular infiltration	
PBS -1	++	++	+	+		+	moderate
PBS -2	+	+	+	+		+	mild
PBS -3	+	+	+	+		+	mild
MR3-MR3-ABD -1	+		+	+			mild
MR3-MR3-ABD -2							normal
MR3-MR3-ABD -3	+		+	+			mild

Supplementary Fig. 12. Lung viral loads and histopathology from infected mice at 3 dpi. (**a**, **b**) Lung viral loads as determined by PCR (**a**) and $TCID_{50}$ assay (**b**). Error bars represent the standard error of the mean (SEM, n=3 biologically independent samples). Raw data were transformed to approximate normal distribution before statistical analysis using unpaired, two-tailed student's *t*-test. ns, not significant. ***, $p = 0.0009$. (**c**) Histopathology of lungs from the three mice in the PBS group. (**d**) Histopathology of lungs from the three mice from the MR3-MR3-ABD group. Arrows denote inflammatory cell infiltration. A black triangle indicates a typical red blood cell exudation, and a blue triangle indicates typical compensatory expansion of the alveolar cavity. The left panels denote an overview of the lung at 10× magnification. The right panels denote the expanded view of the black boxes in the left panels, at 100× magnification. Bars = 100 μm. (**e**) Scoring of the data in **c** and **d**. ABD, albumin-binding domain; PBS, phosphate-buffered saline. Source data and statistical information for **a** and **b** are provided as a Source Data file.

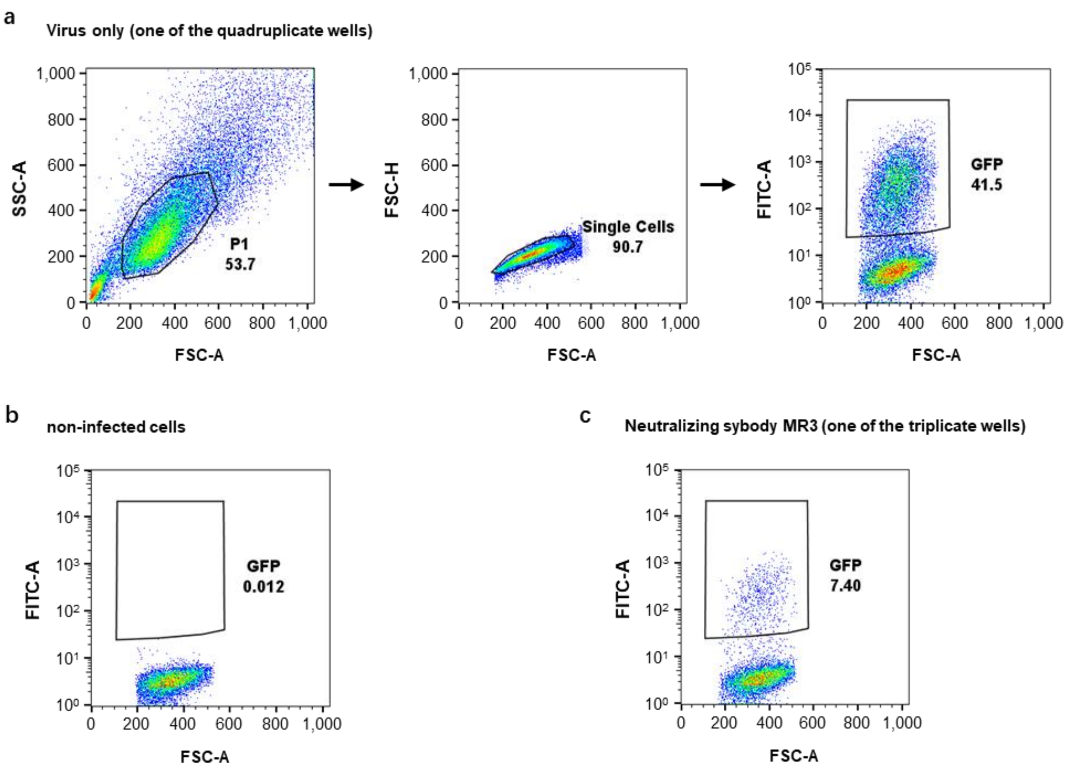

Supplementary Fig. 13. Representative flow cytometry gating strategy for neutralization assay. (**a**) Gating strategy and FACS plots of cells infected with virus. (**b**) FACS plots of non-infected cells. (**c**) FACS plots of infected cells treated with sybody MR3 at 1 μM concentration. In **a** and **c**, cells were infected with MLV/SARS-CoV-2 pseudotyped particles transducing a GFP-expressing cassette. Therefore, the expression level of the GFP reporter in single cells is an indication for infection. The percentage of GFP-positive cells in **c** was normalized to that of the non-infected and virus-only cells before calculation of neutralizing activity. FACS data are related to Supplementary Fig. 3a (*iii*). FACS, fluorescence-activated cell sorting; FSC-A/H, forward scatter area/height; FITC, fluorescein isothiocyanate (channel used in FACS analysis); GFP, green fluorescence protein; P1, population 1 (live cells); SSC-A/H, side scatter area/height.

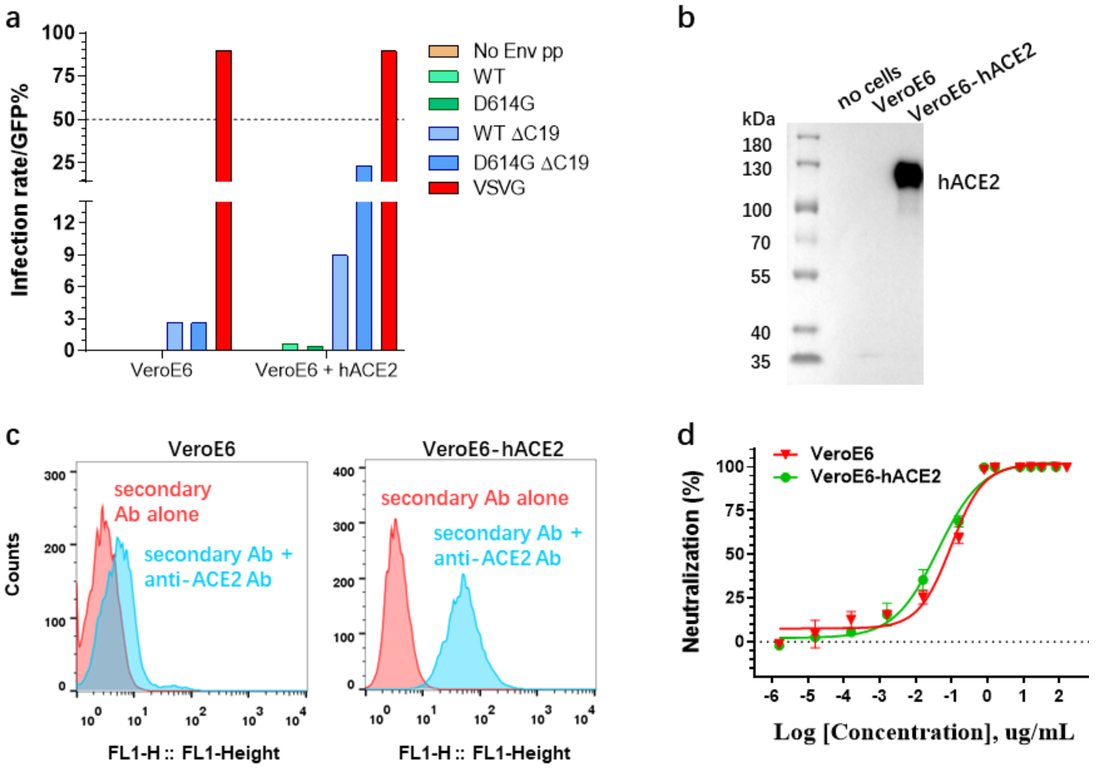

Supplementary Fig. 14. Characterization of VeroE6 cells overexpression human angiotensin-converting enzyme 2 (hACE2). (**a**) Comparative transduction efficiencies by different SARS-CoV-2 pseudotyped particles (pp) on VeroE6 and VeroE6-ACE2. Cells were seeded in a 48-well plate (10^4 cells/well) and transduced 24 h latter using pp harbouring S from wild-type SARS-CoV-2 (WT, Wuhan strain, cyan) or S from the D614G mutant (D614G, green), or C-terminal (19 amino acids) truncated S from WT (WT ΔC19; Wuhan strain, light blue) or the ΔC19 on the D614G mutant (D614G ΔC19, blue). Pseudotyped particles with no envelope glycoprotein at its surface (No Env pp, orange) serve as a negative control and G glycoprotein of the vesicular stomatitis virus (VSVG) enveloped lentivirus pp (red) serve as a positive control. A representative experiment is presented. (**b**) Validation of hACE2 overexpression in VeroE6 cells by Western blotting. Expression of human ACE2 of VeroE6 cells after transduction using a lentivirus vector and selection using puromycine. Cell lysate was analysed by Western blotting using a rabbit anti-ACE2 antibody (Cat. 10108-T56, Sinobio, China; 1:2,000 dilution) and a conjugated anti rabbit-HRP secondary antibody (Cat A0208, Beyotime, China; 1:1,000 dilution). (**c**) Validation of hACE2 expression on the cell surface by flow cytometry. Cells were detached using EDTA, wash in PBS, and analysed by flow cytometry using a rabbit anti-ACE2 antibody (same as above) and an Alexa488-conjugated secondary antibody (Cat A11034, Life Technologies, 1:500 dilution). Cells incubated with secondary antibody alone were analysed to assess non-specific binding. A representative experiment is presented. (**d**) Comparison of Fc-MR3 IC_{50} on naïve VeroE6 and VeroE6-hACE2 cells. Neutralization assays using dose response of Fc-MR3 antibody were conducted. The % of inhibition compare to the 100% maximum infection without Fc-MR3 are plotted and IC_{50} measured (VeroE6, 0.09 μg/mL; VeroE6-hACE2, 0.04 μg/mL) using regression. Mean ± standard deviation are plotted (n=3 independent experiments). Source data for **d** are provided as a Source Data file.

References

1 Baker, N. A., Sept, D., Joseph, S., Holst, M. J. & McCammon, J. A. Electrostatics of nanosystems: Application to microtubules and the ribosome. *Proc Natl Acad Sci USA* **98**, 10037-10041, doi:10.1073/pnas.181342398 (2001).

2 Walls, A. C. *et al.* Structure, Function, and Antigenicity of the SARS-CoV-2 Spike Glycoprotein. *Cell* **181**, 281-292.e286, doi:10.1016/j.cell.2020.02.058 (2020).

3 Zimmermann, I. *et al.* Synthetic single domain antibodies for the conformational trapping of membrane proteins. *eLife* **7**, e34317, doi:10.7554/eLife.34317 (2018).
