# Supplementary material for: A synthetic nanobody targeting RBD protects hamsters from SARS-CoV-2 infection
Source: Nat Commun. 2021 Jul 30;12:4635. doi: 10.1038/s41467-021-24905-z (PMC8324831; doi:10.1038/s41467-021-24905-z)
Supplement: Supplementary file 3 — Description of Additional Supplementary Files [file 41467_2021_24905_MOESM3_ESM.docx]

**Description of Additional Supplementary Files**

**Title: Supplementary Data 1.**

**Description:** Sequences and fluorescence-detection size exclusion chromatography results of sybodies gainst the SARS-CoV-2 Spike receptor-binding domain.

**Title Supplementary Data 2.**

**Description:** Primers and sequences used in this study.
