# Supplementary material for: A synthetic nanobody targeting RBD protects hamsters from SARS-CoV-2 infection
Source: Nat Commun. 2021 Jul 30;12:4635. doi: 10.1038/s41467-021-24905-z (PMC8324831; doi:10.1038/s41467-021-24905-z)
Supplement: Supplementary file 6 — Reporting Summary [file 41467_2021_24905_MOESM6_ESM.pdf]

nature portfolio

Corresponding author(s): Dianfan Li, Dimitri Lavillette, Yuhai Bi, Gary Wong

Last updated by author(s): Jul 6, 2021

Reporting Summary

Nature Portfolio wishes to improve the reproducibility of the work that we publish. This form provides structure for consistency and transparency in reporting. For further information on Nature Portfolio policies, see our Editorial Policies and the Editorial Policy Checklist.

Statistics

For all statistical analyses, confirm that the following items are present in the figure legend, table legend, main text, or Methods section.

n/a	Confirmed	
☐	☒	The exact sample size (n) for each experimental group/condition, given as a discrete number and unit of measurement
☐	☒	A statement on whether measurements were taken from distinct samples or whether the same sample was measured repeatedly
☐	☒	The statistical test(s) used AND whether they are one- or two-sided<br>*Only common tests should be described solely by name; describe more complex techniques in the Methods section.*
☒	☐	A description of all covariates tested
☐	☒	A description of any assumptions or corrections, such as tests of normality and adjustment for multiple comparisons
☐	☒	A full description of the statistical parameters including central tendency (e.g. means) or other basic estimates (e.g. regression coefficient) AND variation (e.g. standard deviation) or associated estimates of uncertainty (e.g. confidence intervals)
☐	☒	For null hypothesis testing, the test statistic (e.g. F, t, r) with confidence intervals, effect sizes, degrees of freedom and P value noted<br>*Give P values as exact values whenever suitable.*
☒	☐	For Bayesian analysis, information on the choice of priors and Markov chain Monte Carlo settings
☒	☐	For hierarchical and complex designs, identification of the appropriate level for tests and full reporting of outcomes
☒	☐	Estimates of effect sizes (e.g. Cohen's d, Pearson's r), indicating how they were calculated

Our web collection on statistics for biologists contains articles on many of the points above.

Software and code

Policy information about availability of computer code

Data collection	Absorbance, Softmax Pro 7.0; Fluorescence-detection size exclusion chromatography, Lab Solutions 5.87; Preparative size exclusion chromatography, ChromLab 3.3.0.09; Bio-layer interferometry, Octet RED96 system; Crystal imaging, RockImager 3.4.3.1; X-ray diffraction, Blu-Ice; Fluorescence activated Cell Sorting, CellQuest Pro; Hydrogen-deuterium exchange mass spectrometry, Thermo LTQ Orbitrap-Elite mass spectrometer; Cryo-EM data collection, SerialEM.
Data analysis	Binding kinetics, Analysis 10.0; Curve fitting for the neutralization data and animal data analysis, GraphPad Prism 8.4.2.679; The FACS data analysis, FlowJo; Hydrogen-deuterium exchange mass spectrometry, HDExaminer 2.0 (Sierra Analytics Inc., Modesto, CA);X-ray diffraction data processing, XDS Version January 31, 2020; X-ray diffraction data scaling and molecular replacement, Aimless/Phaser in ccp4 7.0.078; 3D-model building, Coot 0.8.9.2; Structure refinement, Phenix 1.9-1692; Structure visualization, PyMOL 2.3.3. Cryo-EM data analysis, Relion3.1, MotionCor2, CTFFIND4, cryoSPARC; Cryo-EM map segmentation, UCSF Chimera and ChimeraX. Curve presentation of the FSEC and BLI results, OriginPro 9.6.0.172. ELISA result analysis, Microsoft Excel Standard 2013

For manuscripts utilizing custom algorithms or software that are central to the research but not yet described in published literature, software must be made available to editors and reviewers. We strongly encourage code deposition in a community repository (e.g. GitHub). See the Nature Portfolio guidelines for submitting code & software for further information.

Data

Policy information about availability of data

All manuscripts must include a data availability statement. This statement should provide the following information, where applicable:

- Accession codes, unique identifiers, or web links for publicly available datasets
- A description of any restrictions on data availability
- For clinical datasets or third party data, please ensure that the statement adheres to our policy

The structure factors and coordinates are available through the protein data bank (PDB) under accession codes 7C8V (SR4-RBD), 7C8W (MR17-RBD), and 7CAN (MR17-K99Y in complex with the RBD). The cryo-EM map of MR3-Spike has been deposited to EMDB with accession ID of EMD-31328. The raw data for Figures 1b-2d, 2g, 2h, 3b-3j, 4a-4d, 5a-5j, and the statistical analysis of Fig. 5c-5k are available as supplementary datasets.

Field-specific reporting

Please select the one below that is the best fit for your research. If you are not sure, read the appropriate sections before making your selection.

[x] Life sciences [ ] Behavioural & social sciences [ ] Ecological, evolutionary & environmental sciences

For a reference copy of the document with all sections, see nature.com/documents/nr-reporting-summary-flat.pdf

Life sciences study design

All studies must disclose on these points even when the disclosure is negative.

Sample size	The sample size for mice in the live viruses work was 3. This small number was chosen merely because of very limited BSL3 resources available to us. As a preliminary test we proceeded with the 3 mice in each group.<br>The sample size for hamster experiments was 5 which would allow proper statistical analysis.<br>The sample size for in vivo stability assay of sybodies in mice was set at 2, as a somewhat preliminary test.<br>The sample size for toxicity assay of the sybodies in mice was 4.
Data exclusions	No data was excluded in the analyses.
Replication	The neutralization assays were performed in three or four independent experiments except for Fig. 4c, one sample (MR3-MR3-34GS against 614G) in Fig. 4d, sample 614D in Fig. 5a, Supplementary Fig. 3, and Supplementary 10a/10b of which the data are the representative from two independent experiments.<br><br>The animal studies were not repeated.
Randomization	Randomization was performed while assigning the animals into experimental groups.
Blinding	The pathological analysis was performed by experts who did not know the identity of the experimental groups. Other experimental results were analyzed without blinding as the readout are considered to be not influenced by the prior knowledge.

Reporting for specific materials, systems and methods

We require information from authors about some types of materials, experimental systems and methods used in many studies. Here, indicate whether each material, system or method listed is relevant to your study. If you are not sure if a list item applies to your research, read the appropriate section before selecting a response.

Materials & experimental systems

n/a	Involved in the study	
[ ]	[x]	Antibodies
[ ]	[x]	Eukaryotic cell lines
[x]	[ ]	Palaeontology and archaeology
[ ]	[x]	Animals and other organisms
[x]	[ ]	Human research participants
[x]	[ ]	Clinical data
[ ]	[x]	Dual use research of concern

Methods

n/a	Involved in the study	
[x]	[ ]	ChIP-seq
[ ]	[x]	Flow cytometry
[x]	[ ]	MRI-based neuroimaging

Antibodies

Antibodies used	Polyclonal rabbit anti-SARS-CoV-2 antibodies (Cat. 40589-T62, Sino Biological, Chesterbrook, PA, USA; 1:1,000 dilution);

Goat anti-rabbit IgG conjugated with the horseradish peroxidase (Cat. P0448, DAKO, Agilent Technologies Neterlands, The Neterlands; 1:100 dilution);
Monoclonal Anti-c-Myc antibody produced in mouse (Cat. M4439, Sigma, USA; 1:2,000 dilution)
Polyclonal rabbit anti-ACE2 antibodies (Cat. 10108-T56, Sinobio, China; 1:2,000 dilution)
Goat anti-rabbit IgG (H+L)-HRP (Cat. A0208, Beyotime, China; 1:1,000 dilution)
Alexa Fluor 488 Goat Anti-Rabbit IgG(H+L)-FITC (Cat. A11034, Life technologies, USA; 1:500 dilution)

Validation

All the antibodies (except for mAbs and nanobodies against SARS-CoV-2 S-RBD which are produced in house) in this study are commercial antibodies, and the validation were conducted by the manufacturer.

According to the manufacturer's website, "polyclonal anti-SARS-CoV-2 antibody (from rabbit, Cat. 40589-T62) is produced in rabbits immunized with purified, recombinant SARS-CoV-2 / 2019-nCoV Spike Protein (Catalog#40589-V08B1). It reacts with SARS-CoV-2 Coronavirus spike using ELISA and Western Blotting and cross reacts with SARS-CoV Spike S1 (Cat# 40150-V08B1), SARS-CoV Spike RBD (Cat# 40150-V08B2),
SARS-CoV-2 (2019-nCoV) Spike S1 (Cat# 40591-V08H), and SARS-CoV-2 (2019-nCoV) Spike RBD (Cat# 40592-V05H)."

Goat anti-rabbit IgG conjugated with the horseradish peroxidase. "The cross-reaction with human, mouse and rat immunoglobulins, and fetal calf serum proteins is negligible. The goat antibody used for horseradish peroxidase-conjugation reacts with rabbit immunoglobulins of all classes." The product link is https://www.citeab.com/antibodies/3288347-p0448-goat-anti-rabbit-immunoglobulins-hrp-affinity.

Monoclonal Anti-c-Myc antibody produced in mouse. The antibody production clone is 9E10, monoclonal. And the information of the product is linked to https://www.sigmaaldrich.cn/certificates/COFA/M4/M4439/M4439-BULK________029M4849V_.pdf
The antibody is verified using western blot analysis with E.coli expressing c-Myc tag. The IgG concentration is 2.3 mg/mL and the western blot titer is 1:7,000.

Polyclonal rabbit anti-ACE2 antibodies is produced in rabbits immunized with recombinant Human ACE2 / Angiotensin-Converting Enzyme 2 Protein. According to the manufacturer's information, it can be used in ELISA, Western Blotting and IHC-P.

According to the website information, goat anti-rabbit IgG (H+L)-HRP can be used for western blotting analysis as 1:1,000 dilution; ELISA analysis as 1:250 dilution; and IHC as 1:50 dilution.

Alexa Fluor 488 Goat Anti-Rabbit IgG(H+L)-FITC. The polyclonal antibody is produced in goat immunized with Gamma Immunoglobins Heavy and Light chains. The purified antibody is conjugated with Alexa Fluor® 488. The antibody concentration is 2 mg/mL and according to the test, 1-10 ug/mL labeled antibody can be used for flow cytometry.

The validation of the SARS-CoV-2 neutralizing monoclonal antibodies (REGN10933, REGN10987, CV30, and CB6) were carried out in the study as they bound to RBD (Fig. 3c-3f) and in the literature cited in the paper where they are mentioned.

The nanobodies are synthetic nanobodies with known sequences (and as listed in Supplementary Table 1).

Eukaryotic cell lines

Policy information about cell lines

Cell line source(s)	Commercial sources for the cell lines are: Trichoplusia ni High Five insect cells, Thermofisher Cat. B85502; sf9 insect cells, Thermofisher Cat. B82501; HEK293T cells, ATCC Cat. CRL-3216; Expi293 cells, ThermoFisher Cat. A14527; VeroE6 cells, ATCC Cat. CRL-1586; Pichia pastoris GS115, ThermoFisher Cat. C18100; Pichia Pastoris SMD1168H, ThermoFisher Cat. C18400.
Authentication	No
Mycoplasma contamination	Cell lines were not tested for mycoplasma contamination.
Commonly misidentified lines (See ICLAC register)	No commonly misidentified cell lines were used in the study.

Animals and other organisms

Policy information about studies involving animals; ARRIVE guidelines recommended for reporting animal research

Laboratory animals

For toxicity assays:
Female ICR (CD-1) mice (7-weeks old) were kept in the SPF (specific pathogen free) animal facility with controlled temperature (24 °C, range: 20-26 °C), humidity (69%, range: 40-70%), and lighting conditions (12 h light/12 h dark cycle).

For live virus challenge experiments:
C57BL/6J female mice (aged 6-8 weeks) were socially housed (2-3 mice per filter top cage), placed in Class III isolator, under controlled conditions of humidity (57.5%, range: 54-61%), temperature (22 °C, range: 21-23 °C), and light (12-hour light/12-h dark

cycle).

For live virus challenge experiments:
Female Syrian golden hamsters (Mesocricetus auratus; strain RjHan:AURA, purpose bred from Janvier, France, 6-weeks old) were socially housed (2-3 animals per filter top cage, (T3, Techniplast), placed in Class III isolators, under controlled conditions of humidity (55%, range 50-60%), temperature (21 °C, range: 19-23 °C), and light (12-hour light/12-hour dark cycles).

Wild animals	No
Field-collected samples	No
Ethics oversight	The mice experiments for in vivo stability were approved by the Institutional Animal Care and Use Committee of the Institut Pasteur of Shanghai, Chinese Academy of Sciences (Animal protocol No. A2020009) for in vivo stability assays.<br><br>The mice experiments with live SARS-CoV-2 were approved by the Ethics Committees of Institute of Microbiology, Chinese Academy of Sciences (SQIMCAS2020010). The mice study was conducted in strict accordance with the recommendations provided in the Guide for the Care and Use of Laboratory Animals of the Ministry of Science and Technology of the People's Republic of China.<br><br>For hamsters, animals were handled in an ABSL3 biocontainment laboratory. The research was conducted in compliance with the Dutch legislation for the protection of animals used for scientific purposes (2014, implementing EU Directive 2010/63) and other relevant regulations. The licensed establishment where this research was conducted (Erasmus MC) has an approved OLAW Assurance # A5051-01. The research was conducted under a project license from the Dutch Central Commission on Animal experiments (CCD) and the study protocol (#17-4312) was approved by the institutional Animal Welfare Body. Animals were socially housed (2-3 animals per filter top cage, (T3, Techniplast), placed in Class III isolators, under controlled conditions of humidity, temperature, and light (12-hour light/12-hour dark cycles). Food and water were available ad libitum. Animals were cared for and monitored (pre- and post-infection) by qualified personnel. The animals were sedated/anesthetized for all invasive procedures.

Note that full information on the approval of the study protocol must also be provided in the manuscript.

Dual use research of concern

Policy information about dual use research of concern

Hazards

Could the accidental, deliberate or reckless misuse of agents or technologies generated in the work, or the application of information presented in the manuscript, pose a threat to:

No	Yes	
☒	☐	Public health
☒	☐	National security
☒	☐	Crops and/or livestock
☒	☐	Ecosystems
☒	☐	Any other significant area

Experiments of concern

Does the work involve any of these experiments of concern:

No	Yes	
☒	☐	Demonstrate how to render a vaccine ineffective
☒	☐	Confer resistance to therapeutically useful antibiotics or antiviral agents
☒	☐	Enhance the virulence of a pathogen or render a nonpathogen virulent
☒	☐	Increase transmissibility of a pathogen
☒	☐	Alter the host range of a pathogen
☒	☐	Enable evasion of diagnostic/detection modalities
☒	☐	Enable the weaponization of a biological agent or toxin
☒	☐	Any other potentially harmful combination of experiments and agents

Flow Cytometry

Plots

Confirm that:

- [x] The axis labels state the marker and fluorochrome used (e.g. CD4-FITC).
- [x] The axis scales are clearly visible. Include numbers along axes only for bottom left plot of group (a 'group' is an analysis of identical markers).
- [x] All plots are contour plots with outliers or pseudocolor plots.
- [x] A numerical value for number of cells or percentage (with statistics) is provided.

Methodology

Sample preparation	Cells were rinced with PBS, trypsinized from 48-well plates, and then resuspended in 4% PFA.
Instrument	BD FACS Celesta Flow Cytometer, BD Biosciences.
Software	FlowJo TM
Cell population abundance	No post-sort fractions were collected.
Gating strategy	Cells were first gated by FSC and SSC to obtain single cells, and then gated for GFP Positive (infected) versus Negative (uninfected).

- [x] Tick this box to confirm that a figure exemplifying the gating strategy is provided in the Supplementary Information.
